# Evidence for an ancient aquatic origin of the RNA viral order *Articulavirales*

**DOI:** 10.1073/pnas.2310529120

**Published:** 2023-10-31

**Authors:** Mary E. Petrone, Rhys Parry, Jonathon C. O. Mifsud, Kate Van Brussel, Ian Vorhees, Zoe T. Richards, Edward C. Holmes

**Affiliations:** ^a^Sydney Institute for Infectious Diseases, School of Medical Sciences, The University of Sydney, Sydney, NSW 2006, Australia; ^b^Laboratory of Data Discovery for Health Limited, Hong Kong Special Administrative Region, China; ^c^School of Chemistry and Molecular Biosciences, The University of Queensland, Brisbane, QLD 4067, Australia; ^d^James A. Baker Institute for Animal Health, Department of Microbiology and Immunology, College of Veterinary Medicine, Cornell University, Ithaca, NY 14850; ^e^Coral Conservation and Research Group, Trace and Environmental DNA Laboratory, School of Molecular and Life Sciences, Curtin University, Perth, WA 6102, Australia; ^f^Collections and Research, Western Australian Museum, Welshpool, WA 6106, Australia

**Keywords:** Articulavirales, influenza, Cnidaria, evolution, virosphere

## Abstract

The emergence of previously unknown disease-causing viruses in mammals is in part the result of a long-term evolutionary process. Reconstructing the deep phylogenetic histories of viruses helps identify major evolutionary transitions and contextualizes the emergence of viruses in new hosts. We used a combination of total RNA sequencing and transcriptome data mining to extend the diversity and evolutionary history of the RNA virus order *Articulavirales*, which includes the influenza viruses. We identified instances of *Articulavirales* in the invertebrate phylum Cnidaria (including corals), constituting a novel and divergent family that we provisionally named the “Cnidenomoviridae.” We further extended the evolutionary history of the influenza virus lineage by identifying four divergent, fish-associated influenza-like viruses, thereby supporting the hypothesis that fish were among the first hosts of influenza viruses. In addition, we substantially expanded the phylogenetic diversity of quaranjaviruses and proposed that this genus be reclassified as a family—the “Quaranjaviridae.” Within this putative family, we identified a novel arachnid-infecting genus, provisionally named “Cheliceravirus.” Notably, we observed a close phylogenetic relationship between the Crustacea- and Chelicerata-infecting “Quaranjaviridae” that is inconsistent with virus–host codivergence. Together, these data suggest that the *Articulavirales* has evolved over at least 600 million years, first emerging in aquatic animals. Importantly, the evolution of the *Articulavirales* was likely shaped by multiple aquatic–terrestrial transitions and substantial host jumps, some of which are still observable today.

## Significance

Viruses that cause disease in humans comprise a tiny fraction of global virus diversity. Determining the long-term evolutionary history of disease-causing virus lineages places the events that drive virus emergence in an evolutionary context. Understanding when and from where viral lineages originate, and how often they jump species boundaries to emerge in new hosts, is central to this process. We addressed these questions with respect to the RNA virus order *Articulavirales* which contains the vertebrate influenza viruses. Our metagenomic and phylogenetic study doubled the number of known *Articulavirales* families, revealed a complex interplay of virus–host codivergence and cross-species virus transmission over deep evolutionary time, and traced the order’s likely origins to ancient aquatic animals at least 600 Mya.

Zoonotic viruses transmitted from other animals to humans have caused multiple epidemics in recent decades (1, 2), and the frequency of these events is projected to increase as a consequence of a myriad of reasons including climate change (3). To combat this threat research on zoonotic risk aims to identify viruses that are primed to spill over into humans. It is commonly assumed that the viruses most likely to cause future epidemics are genetically similar to those that have previously caused outbreaks (4, 5). A range of field-based and bioinformatic methods aimed at detecting potentially zoonotic viruses are predicated on this assumption (6–8). Although these approaches help construct a picture of virus diversity, the process of cross-species virus transmission that drives disease emergence in the short term also plays a key role in virus speciation in the long term, shaping virus–host associations that likely date back to the existence of single-celled organisms (9). In addition to revealing their antiquity, elucidating the deep evolutionary history of known disease-causing viruses provides important context for understanding the true rate at which viruses jump species boundaries to infect new hosts.

Because they reveal the viromes of a diverse array of organisms, metagenomic data are a powerful tool for tracing long evolutionary histories (10). These data have already shown that the global virosphere is vast and largely unexplored (10), and the recent exploration of marine environments has demonstrated that the ocean is a rich source of virus diversity (11). Metagenomic research has also shown that viruses once thought to be restricted to mammals in reality exist in a wide variety of other vertebrates (12), suggesting that they have long evolutionary histories in animals. Similarly, disease-causing lineages of vertebrate viruses can have origins in invertebrate hosts. For example, flavi-like viruses (single-strand positive-sense RNA viruses) were recently identified in ancient invertebrates (Cnidaria) (13), basal chordates (Ascidia) (13), and in diverse aquatic environments (14). Collectively, this suggests that flavi-like viruses and their relatives, which include such human pathogens as dengue virus and yellow fever virus, emerged concurrently with the origin of the Metazoa (i.e., animals) some 750 to 800 Mya (15). Indeed, that aquatic ecosystems may have harbored the ancestors of terrestrial viruses speaks to their antiquity and the biological diversity that these environments support.

The order *Articulavirales* is of special importance in exploring the intersection of marine life and the evolution of disease-causing viruses. Viruses within the *Articulavirales* have segmented, negative-sense RNA genomes. This order is currently organized into two families—the *Orthomyxoviridae* and the *Amnoonviridae*. The former receives frequent public health attention because it contains three genera that can cause disease in humans: *Thogotovirus*, *Quaranjavirus*, and *Influenzavirus*. Thogotoviruses were first isolated in the 1960s (16), while discoveries of disease-causing quaranjaviruses are ongoing [e.g., the discovery of Wellfleet Bay virus as the cause of avian mortality in 2014 (17)]. Both thogotoviruses and quaranjaviruses periodically spill over into humans, other mammals, and birds through tick-mediated transmission. In contrast, influenza viruses cause seasonal epidemics in human populations, as well as occasional pandemics.

Aquatic animals serve as hosts for viruses in both families. The *Amnoonviridae* is a family of primarily fish-infecting viruses (18), with two species also found in amphibians (19, 20). Tilapia lake virus, a genus within this family, has important implications for the global aquaculture industry because it is associated with severe disease in tilapia (21). Similarly, salmon isavirus (*Orthomyxoviridae*) causes overt disease in salmon (22), and influenza-like viruses have been identified in fish and amphibians (12, 23), although with unknown disease associations. Given their host distribution, the *Amnoonviridae* likely emerged in aquatic animals. However, the long branch lengths associated with this family in current the *Articulavirales* phylogeny suggest substantial unsampled diversity.

Genomic architecture varies within and between the families of the *Articulavirales*, but all contain three polymerase segments: polymerase basic 1 (PB1), polymerase basic 2 (PB2), and polymerase basic 3/polymerase acidic (PB3/PA). These proteins form a heterotrimer comprising the canonical RNA-dependent RNA polymerase (RdRp) (24) that is routinely used as a phylogenetic marker for RNA viruses (25). PB1 contains the palm domain, defined by the SDD amino acid motif, and is therefore the most highly conserved of the subunits. For this reason, PB1 is the segment that has the highest probability of being identified from metagenomic data.

We applied a combination of total RNA sequencing and data mining to revisit the evolutionary history of the *Articulavirales*. To date, no *Articulavirales* genera have been identified in nonbilaterian (i.e., lacking body symmetry) invertebrates of the Cnidaria, which includes corals, jellyfish, and hydra. To address this, we analyzed the RNA viromes of Hexacorals and Octocorals from Australia. Concurrently, we screened the NCBI Transcriptome Shotgun Assemblies (TSA) database for novel orthomyxo-like viruses associated with aquatic animals: Phylum Arthropoda (e.g., shrimp and crabs); Phylum Chordata, Subphylum Vertebrata (fish); Subphylum Tunicata (sea squirts and tunicates); Phylum Mollusca (e.g., squid and whelk); Phylum Porifera (sponges); and Phylum Bryozoa. We combined the resulting datasets to characterize the role of ancient aquatic animals in the evolution of the *Articulavirales* and determine when this order may have emerged in relation to the evolution of the Metazoa.

## Results

### Discovery of Highly Divergent *Articulavirales* in Ancient Invertebrates.

**Fig. 1.**
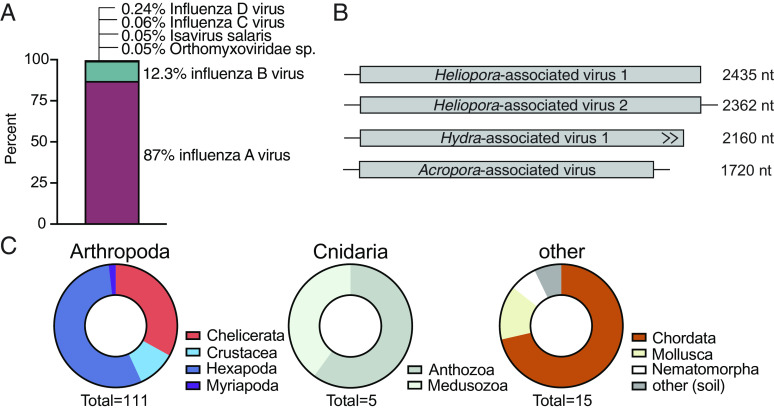
Expanding the known diversity of the *Articulavirales.* (*A*) Overrepresentation of influenza A and B viruses in publicly available genomic data. Distribution of 99.7% of publicly available PB1 segments on NCBI Virus as of January 2023 by virus species. Those virus species not shown each comprise <0.05% of available data. (*B*) PB1 segments of novel *Articulavirales* identified in Cnidaria hosts. *Heliopora*-associated virus 1 was identified through sequencing while *Heliopora*-associated virus 2 was identified through data mining. (*C*) Distribution of host species and classes associated with the *Articulavirales* detected through total RNA sequencing and data mining.

Influenza viruses are overrepresented in publicly available data for viruses in the order *Articulavirales*. Of the 76,887 *Articulavirales* PB1 segments available on NCBI Virus in January 2023, only 291 (0.38%) were not influenza or influenza-like viruses (Fig. 1*A*). Thus, our first objective was to expand the known diversity of noninfluenza viruses in this order.

To search for highly divergent *Articulavirales*, we performed total RNA sequencing on 128 corals (Cnidaria) collected in Western Australia. From these, we identified *Articulavirales* PB1 segments in two libraries—*Heliopora coerulea* (blue coral) (GenBank: OQ939982.1) and *Acropora samoensis* (stony coral) (GenBank: OQ939981.1)—using Diamond BLASTx. Although PB1 is the most conserved polymerase segment, both segments were highly divergent, sharing ≤25% amino acid identity with those of known viruses (*SI Appendix*, Table S1). Neither contained premature stop codons nor returned BLAST hits to known coral genes, suggesting they are not endogenous virus elements (EVEs). However, they were present at very low abundance (both <0.001% non-rRNA reads). The segment recovered from the *Acropora*-associated library (1720nt) was shorter than the *Heliopora*-associated segment (2435nt) (Fig. 1*B*), and the two segments shared minimal sequence similarity to each other (17.8% amino acid pairwise identity). This substantial genetic distance is consistent with the phylogenetic relationship of *Heliopora* and *Acropora* corals. The former are commonly known as blue corals belonging to the Subclass Octocorallia, Order Scleralcyonacea, and estimated to have diverged from other Anthozoa about 500 Mya (26). Acropora, commonly known as staghorn corals, are from the Subclass Hexacorallia, Order Scleractinia, which emerged more recently, likely in the Permian around 260 Mya (26). Analysis of the composition of each library using CCMetagen showed that the majority of eukaryotic genetic material (98%) in the *Acropora* library was associated with Cnidaria (*SI Appendix*, Fig. S1*A*).

In contrast, 73% of contigs assembled from the *Heliopora* library were Suessiales (dinoflagellate symbionts of corals) (*SI Appendix*, Fig. S1*B*). Of the contigs associated with Anthozoa in this library, most (76%) were assigned to Scleractinia rather than *Helioporidae.* This suggested that *Heliporidae* reads were misassigned due to overrepresentation of Hexacorallia in the database (*SI Appendix*, Fig. S1*B*). To test this, we assembled the rRNA contigs identified using sortMeRNA and assessed the host composition against the NCBI nt database. A total of 84.0% of rRNA reads that corresponded to an entry in the nt database were assigned to Octocorallia, with only 4.98% assigned to Hexacorallia (Dataset S1). Assuming that any *Articulavirales* that infect protists such as dinoflagellates would share less than 25% sequence identity to known animal-infecting viruses, the high proportion of reads assigned to Octocorallia suggest that *Heliopora* (Octocorallia) was the most likely host of the virus identified in this library.

We used these segments to screen the Cnidaria assemblies available in the NCBI TSA database (n = 50). This yielded two additional novel PB1 segments: one in a *Heliopora coerulea* coral library (GFVH01041936.1) and another in a *Hydra vulgaris* hydra library (HAAC01043982.1). The second *Heliopora*-associated PB1 segment (2362nt) was slightly shorter than the first (2435nt) and shared 47.6% amino acid pairwise identity. The open reading frame (ORF) of the hydra-associated segment was uninterrupted but incomplete (Fig. 1*B*). PB2 and PB3 segments could not be identified in either.

Having identified *Articulavirales* in basal invertebrates, we reasoned that this viral order likely contains unrealized diversity in other aquatic invertebrate hosts. To address this, we screened TSA libraries for Arthropoda, Porifera, Ascidiacae, Bryozoa, Mollusca, Cnidaria, and fish using an iterative process. Our first input sequence was the PB1 segment of the Wenling hagfish influenza-like virus (AVM87635.1) because this was identified in an aquatic host—a jawless fish—and is highly divergent among the *Orthomyxoviridae* in the current *Articulavirales* phylogeny. This search yielded 30 novel (i.e., sharing ≤80% amino acid similarity to the closest BLASTp hit) PB1 segments of at least 1000nt in length. We then used each of these segments as input into the same query, and continued until no additional new sequences could be recovered. Through this process, we detected 94 novel PB1 segments of at least 1000nt in length. To supplement this dataset, we evaluated all RdRp contigs assembled from the Serratus project with an assigned “unknown” *Articulavirales* RdRp palmprint as per the palmDB database (27). After removing known viruses, duplicated sequences, and fragments less than 800nt in length, we identified 35 additional novel viruses.

In total, we discovered 131 novel *Articulavirales* PB1 segments. Most of these viral sequences were associated with arthropod hosts (n = 111), which likely reflects a sampling bias toward Arthropoda rather than the true host distribution of *Articulavirales* (Fig. 1*C* and Dataset S2).

Previous studies have presented evidence for the endogenization of *Orthomyxoviridae* polymerase segments (28). We therefore assessed whether the contigs identified here could be EVEs. PB1 candidates that included premature stop codons were excluded from our dataset. We screened each segment for the presence of host genes using CheckV (29), and none were identified. Because the presence of multiple virus segments in a library would be strongly indicative of exogenous expression, we screened each TSA library in which we had found a PB1 segment for additional virus segments. We recovered three polymerase segments for 91 of the novel viruses we identified, and for 73 and 81 of these, we successfully retrieved nucleoprotein and hemagglutinin segments, respectively (Dataset S2). Our inability to detect additional polymerase segments in the invertebrate libraries using both sequence- and structure-based approaches was likely due to very low similarity of these segments to those of any known virus.

### The *Articulavirales* Comprises at least Four Families and Likely Originated in Ancient, Aquatic Animals.

Given the low sequence similarity of the PB1 segments we identified in the Cnidaria libraries, we hypothesized that these viruses would be phylogenetically distinct from other families in the order. To test this, we aligned the novel 131 sequences identified in this study with a representative sample of publicly available *Articulavirales* PB1 sequences (Dataset S3) using MUSCLE (30) and inferred a maximum likelihood phylogeny using IQ-Tree (31).

**Fig. 2.**
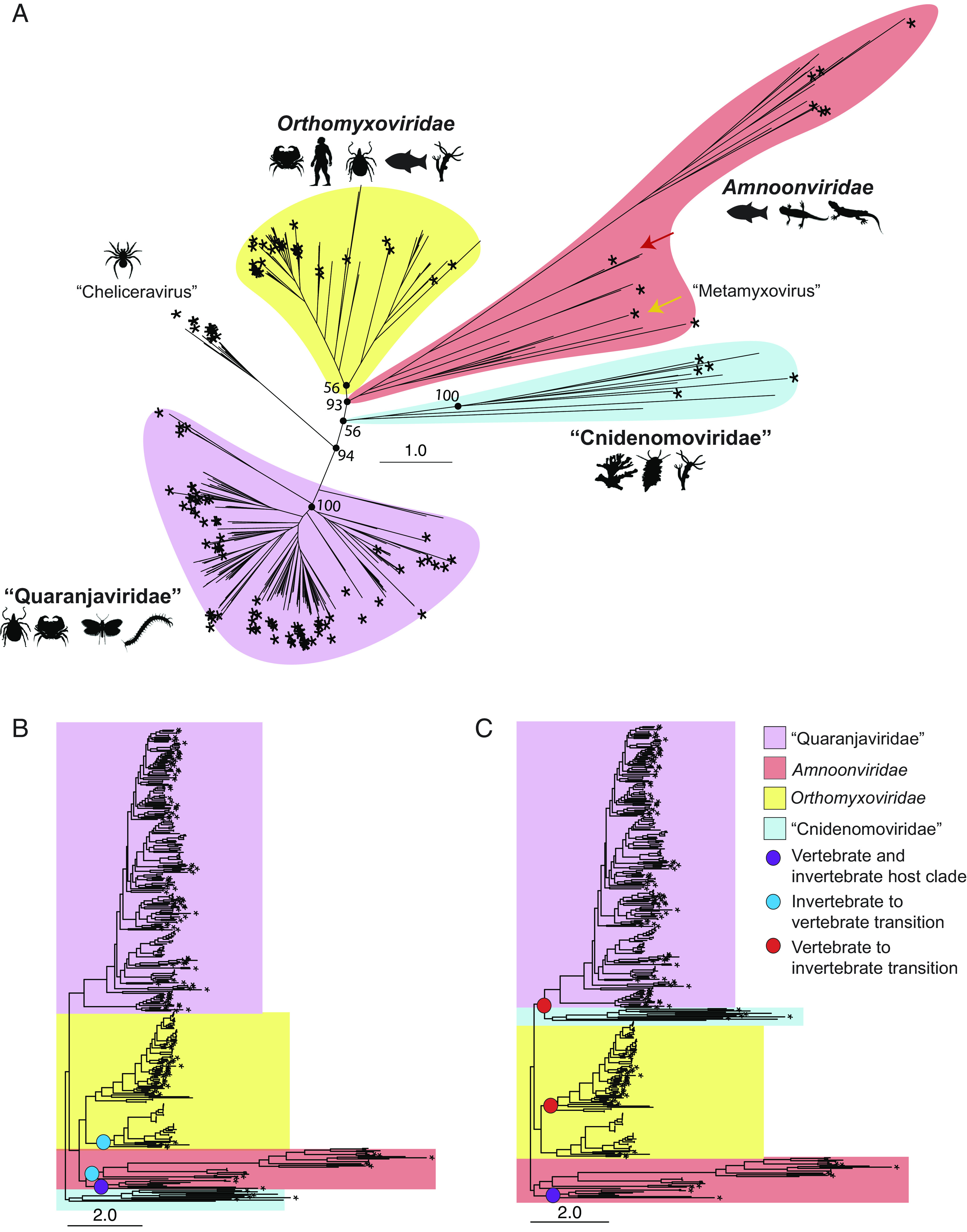
Phylogenetic evidence for four virus families within the order *Articulavirales*. (*A*) Unrooted maximum likelihood tree of the *Articulavirales* PB1 segment, with branch lengths scaled to the number of amino acid substitutions per site. *Novel viruses identified in this study. The red arrow marks Salmon isavirus while the gold arrow marks an aquatic amnoon-like virus clade associated with Mollusca and fish hosts (“Metamyxovirus”). (*B*) PB1 maximum likelihood tree rooted on basal invertebrate family “Cnidenomoviridae”. (*C*) PB1 maximum likelihood tree rooted on the *Amnoonviridae*. In all cases, branch lengths are scaled to the number of amino acid substitutions per site.

The inclusion of the viruses identified here had a substantial impact on the phylogenetic structure of this order. The Cnidaria-associated viruses formed a putative new family currently comprising nonarthropod invertebrate hosts. We have provisionally named this family the “Cnidenomoviridae.” Although there is generally strong support within the “Cnidenomoviridae” (*SI Appendix*, Fig. S2), the phylogenetic position of this family within the *Articulavirales* is uncertain (ufboot = 56). The known hosts of this family are currently coral, hydra, tunicate (Urochordata), sea cucumber (Echinodermata), sea slug (Mollusca), and mussel (Mollusca) (32) (Fig. 2*A*, “Cnidenomoviridae”). Viruses associated with *Heliopora coerulea* corals were more closely related to each other than to the virus identified in the *Acropora* coral, tentatively suggesting class specificity (*SI Appendix*, Fig. S2). This relationship also suggests that *Heliopora* is the host of the *Heliopora*-associated virus we identified through sequencing despite the library composition.

The addition of the arthropod-associated viruses identified through our TSA and palmDB screens suggested that the genus *Quaranjavirus* and the quaranja-like viruses should be reclassified as a new family that we have provisionally denoted as the “Quaranjaviridae” (Fig. 2*A*). We also identified a clade of quaranja-like viruses associated with spiders (Arthropoda, Chelicerata) (Fig. 2*A*, “Cheliceravirus”). Although the PB1 segments of this putative new genus were arguably sufficiently divergent from the “Quaranjaviridae” to constitute a distinct family, this was not true for the other segments, and we discuss this in more detail below. The new tree topology also supported the addition of a genus (Isavirus) to the *Amnoonviridae* through the reclassification of salmon isavirus, which is currently classified as *Orthomyxoviridae* (Fig. 2*A*, red arrow). In sum, we propose that *Articulavirales* comprises at least four families: *Orthomyxoviridae*, *Amnoonviridae*, “Quaranjaviridae”, and “Cnidenomoviridae”. We adopt this new nomenclature for the remainder of the manuscript.

Putative host associations throughout the order are inconsistent with strict virus–host codivergence. For example, viruses associated with Mollusca and Cnidaria hosts did not belong exclusively to the “Cnidenomoviridae”. In particular, we identified amnoon-like viruses associated with a squid (*Sepioloidea lineolate*) and a hydra (GHUC01003666.1) that formed a clade with viruses associated with fish and whelk (Mollusca) (Fig. 2*A*, gold arrow, “Metamyxovirus”). The phylogenetic position of this clade, which comprises exclusively aquatic hosts (salmon louse, marine flatworm, lizardfish, squid, whelk, crustacean, gobie, and hydra), had poor support within the *Amnoonviridae* (ufboot = 37) such that the true placement of this putative new genus—“Metamyxovirus”—is uncertain. In the metamyxoviruses there is no clear separation of vertebrate and invertebrate hosts as observed between *Thogotovirus* and *Influenzavirus* and within other families in the *Articulavirales*, although true host associations cannot be verified with metagenomic data alone.

To interpret the evolutionary history of the *Articulavirales*, we considered various possible rooting positions on the phylogeny. The structure of the tree presents ambiguities because both the *Amnoonviridae* and the “Cnidenomoviridae” are defined by long branch lengths, and both are plausible roots. We first assessed whether the branching patterns were an artifact of an alignment error by comparing the topology of trees inferred using the MUSCLE (30) and MAFFT (33) aligners that utilize different algorithms (*SI Appendix*, Fig. S3). No substantive differences in tree topology were observed, thereby suggesting that large sampling gaps persist in these families and account for the branch lengths. Rooting the phylogeny on the novel basal invertebrate family “Cnidenomoviridae” is compatible with long-term virus–host codivergence (Fig. 2*B*). This phylogenetic history means that the *Articulavirales* likely originated in aquatic invertebrates, potentially around 600 Mya when Medusoza and Anthozoa diverged (26). In this scenario, the *Amnoonviridae* form a sister clade to the *Orthomyxoviridae* and the “Quaranjaviridae”. Applying the parsimony criterion, a transition to vertebrate hosts would have occurred twice independently (Fig. 2*B*, blue circles), while a partial transition to include vertebrate hosts would have occurred with the evolution of “Metamyxovirus” (Fig. 2*B*, purple circle).

In contrast, placing the *Amnoonviridae* as the root introduces vertebrate-to-invertebrate transitions, such that the evolution of the order was not shaped by virus–host codivergence (Fig. 2*C*). In this representation of phylogenetic history, the “Cnidenomoviridae” are a sister clade to the “Quaranjaviridae”, and there is a clear division between the invertebrate-only and vertebrate host sections of the tree (the “Metamyxoviruses” notwithstanding). There is also a division between arthropod-associated (the “Quaranjaviridae”) and non-arthropod-associated (the “Cnidenomoviridae”) viruses, although this partitioning may eventually change with additional sampling. Midpoint rooting is relatively consistent with placing the root on the branch leading to the *Amnoonviridae*, except the monophyly of this family is lost (*SI Appendix*, Fig. S4). Despite these important differences, in all three rooting scenarios, the tree structure indicates that the *Articulavirales* first emerged in ancient, aquatic hosts (either fish or marine invertebrates) before spreading to terrestrial animals.

### Fish Were among the Earliest Hosts of Influenza-Like Viruses.

Tracing the evolution of influenza and influenza-like viruses could shed light on the mechanisms by which these viruses emerged in mammals. Through data mining, we identified the PB1 segment of four divergent influenza-like viruses associated with the transcriptomes of fish: a Siberian sturgeon (*Acipenser baerii*, GIPE01045129.1), a flounder (*Paralichthys olivaceus*, SRR8334112), a seahorse (*Hippocampus guttulatus,* SRR1324961), and a grass carp (*Ctenopharyngodon idella*, SRR6475468). In addition to identifying PB1, we successfully recovered four segments (PB2, PB3, NP, and HA) from the sturgeon library, seven segments (PB2, PB3, NP, HA, NA, M1/M2, and NS1/NEP) from the flounder library, five segments from the seahorse library (PB2, PB3, NP, M1/M2, and HEP), and three segments from the grass carp (PB2, PB3, and NP) (*SI Appendix*, Fig. S5). All segments contained complete ORFs.

**Fig. 3.**
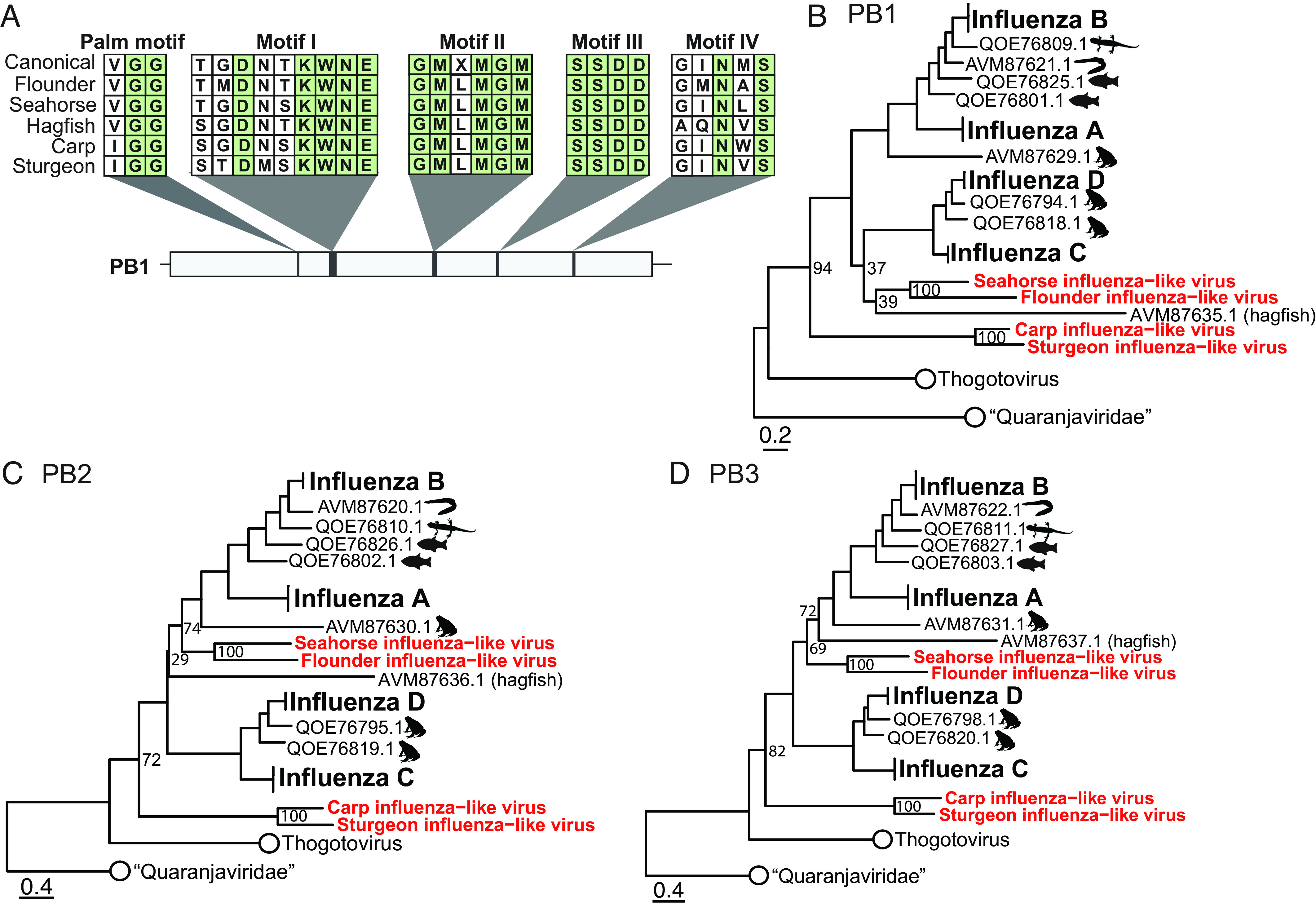
Characterization of a divergent influenza-like viruses identified in a flounder, seahorse, carp, and sturgeon. (*A*) Conserved motifs of the PB1 segment of flounder-, seahorse-, sturgeon-, carp-, and hagfish-associated influenza-like viruses. Maximum likelihood phylogenetic trees of the influenza virus PB1 (*B*), PB2 (*C*), and PB3 (*D*) segments. Putative aquatic hosts are indicated with icons. Segments from viruses identified in this study are shown in red. All trees were rooted using the “Quaranjaviridae” as outgroups with branch lengths scaled to the number of amino acid substitutions per site.

The genomes of the sturgeon- and carp-associated viruses differed from those of canonical influenza viruses, including those discovered in other fish, in two notable ways. First, the predicted protein sequences of the PB2 segment of these viruses are approximately 100 amino acids longer than other segments found in the viruses from this clade (*SI Appendix*, Fig. S5 *A* and *C*). These additional amino acids do not appear to be the result of an insertion as they occur at the 3′ end of the segment. Second, while there is a conserved VGG motif in the palm domain of the PB1 segment of known influenza and thogotoviruses, the PB1 segment of the sturgeon- and carp-associated virus motif is IGG which is common among the “Quaranjaviridae”, found in some members of the “Cnidenomoviridae”, and entirely absent in the *Amnoonviridae* (Fig. 3*A*). The flounder- and seahorse-associated PB1 segments have a VGG motif. Polymerase motifs I-IV were well conserved (Fig. 3*A*). In Motif I, “KWNE” is conserved throughout the clade. The hagfish and our novel influenza-like viruses share identical sequences in motif II. While the SDD sequence in motif III is requisite for functional *Articulavirales* PB1 segments, influenza and influenza-like viruses have a second serine (SSDD) in this motif. Interestingly, motif IV (canonically GINMS) is most divergent in the hagfish virus compared to the sturgeon and carp viruses, but all are characterized by asparagine and serine in the third and fifth positions, respectively.

As the sturgeon transcriptome was generated from the animal’s intestine, we hypothesized that this virus may have been derived from the animal’s diet. To test this, we analyzed the library composition using KMA v1.3.9a (34) and CCMetagen v1.1.3 (35). Interestingly, 64% of all contigs were associated with the host (Actinopteri), and 29.5% aligned to other Chordates (14% Primates, 16.5% Birds, 0.5% *Felidae*) (*SI Appendix*, Fig. S6*A*). Although sturgeon consume other animals in nature, the authors of the study that generated this sequencing library reported that sturgeon were fed commercial fish feed prior to collection (36), rendering the introduction of a competent RNA virus from a different animal species unlikely. The close and consistent phylogenetic relationship of the sturgeon- and carp-associated segments (Fig. 3 *B*–*D*) further supports fish as the bona fide host of these viruses. Thus, given that we were able to recover multiple segments and in the absence of another plausible host, we concluded that the sturgeon was the likely host of this virus.

Phylogenetic analysis of the three polymerase segments revealed that the sturgeon- and carp-associated viruses consistently fall within the influenza clade but are more divergent than all known influenza-like viruses, while the placement of the flounder- and seahorse-associated viruses is less certain (Fig. 3 *B*–*D*). Previously, the most divergent virus in the influenza clade was an influenza-like virus associated with a hagfish (12). However, our analysis found that this placement is dependent on the alignment method. Alignment with MAFFT using the BLOSUM45 and BLOSUM62 scoring matrices recapitulated the reported placement of the hagfish-associated polymerase segments when the novel influenza-like viruses were added to the dataset (*SI Appendix*, Fig. S7). For all three segments, the flounder-, seahorse-, carp-, hagfish-, and sturgeon-associated polymerase segments clustered outside of established species of influenza viruses, with the sturgeon and carp segments consistently comprising the most divergent lineage. Alignment with MUSCLE affected the placement of the hagfish-, seahorse, and flounder-associated PB1 segments, placing them in the same clade albeit with low support (ufboot = 37) (Fig. 3*B*). However, this relationship was better resolved when inferred in the context of the entire order (Fig. 2). Here, the sturgeon- and carp-associated viruses were the most divergent in the clade, followed by the hagfish-associated virus and then the flounder- and seahorse-associated viruses, all with ufboot >95 (*SI Appendix*, Fig. S8). Alignment with MUSCLE also placed the PB2 and PB3 segments of the hagfish-, seahorse-, and flounder-associated viruses as sister lineages to influenza A and B viruses (Fig. 3 *C* and *D*). The polymerase segments of the sturgeon- and carp-associated viruses were consistently the most divergent relative to known influenza-like viruses when either alignment method was used. Moreover, the placement of the three polymerase segments was relatively well supported at the base of the influenza virus clade (ufboot = 94, 72, 82 Fig. 3 *B*–*D*).

To confirm that the unstable placement of the seahorse-, flounder-, and hagfish-associated virus segments was not due to infection by multiple influenza-like strains, we rescreened each library for additional influenza-like segments. All segments presented in Fig. 3 were used as input. No additional segments were found in the libraries, suggesting that the lack of robust support is a consequence of the divergent nature of the viruses rather than to coinfections.

These findings suggest that influenza-like viruses can infect all classes of fish such that these animals may have served as early, if not the first, hosts of influenza-like viruses before their later emergence in birds and mammals.

### Phylogenetic Relationships of “Quaranjaviridae” Segments Suggest a History of Cross-Species Transmission.

Having reasoned that aquatic animals were likely central to the evolution of the *Articulavirales*, we next investigated their role in the evolution of the “Quaranjaviridae”. Nearly all viruses in this family, including those identified in this study, are associated with arthropods as their primary host. While we identified a quaranja-like virus in a horsehair worm (Nematomorpha), as this animal parasitizes arthropods, we assumed that the respective virus was more likely to be associated with an arthropod than with the worm. Previous studies have also identified “Quaranjaviridae” in sediment (14) and feces samples (37), and host associations cannot be determined in these instances. Among the 87 novel “Quaranjaviridae” we identified here, nine were associated with crustaceans (three amphipoda, two crab, and one each for shrimp, squat lobster, copepod, and tanaid).

**Fig. 4.**
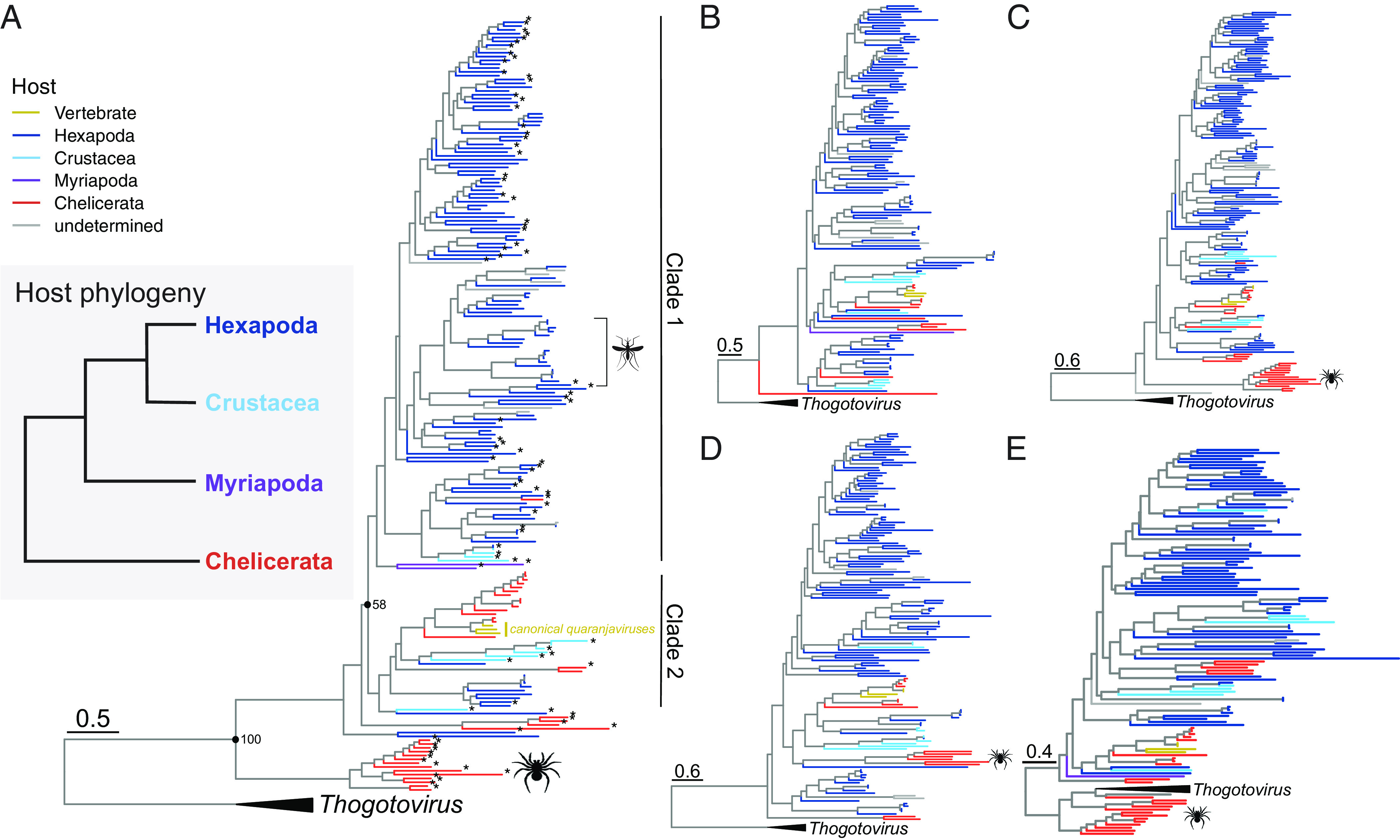
Evolution of the “Quaranjaviridae” is shaped by virus–host codivergence and cross-species transmission. (*A*–*E*) Maximum likelihood phylogenies of the “Quaranjaviridae” rooted with *Thogotovirus*. “Cheliceravirus” are denoted by a spider icon. (*A*), PB2 (*B*), PB3 (*C*), NP (*D*), and HA (*E*). Branch color indicates putative host. Due to limitations in data availability differing numbers of segment were used for each tree (PB1: n = 208; PB2: n = 144; PB3: n = 168; NP: n = 149; HA: n = 128) (*A*) Mosquito-borne viruses are denoted with a mosquito icon. Host phylogeny adapted from Thomas et al. (38) *Novel viruses identified in this study. Branch lengths are scaled according to the number of amino acid substitutions per site.

To evaluate the relationship of these aquatic viruses within the family, we inferred maximum likelihood phylogenetic trees for the three polymerase segments, the nucleoprotein (NP), and hemagglutinin (HA). The composition of these trees necessarily differed as not all segments (particularly NP and HA) were publicly available for known viruses, preventing direct topological comparisons. PB1 segments were available for all viruses, arguably rendering this tree the most reliable. The topology of the PB1 phylogeny revealed both virus–host codivergence and cross-species virus transmission. This phylogeny can be divided into two clades, albeit with limited support (Fig. 4*A*, ufboot = 58). Clade 1 comprises predominantly Hexapoda-associated viruses. However, all four Arthropoda subphyla are represented in this clade, and their relative placement is consistent with the host phylogeny with the exception of one spider-associated virus. In the current host phylogeny, Chelicerata (i.e., ticks, mites, and scorpions) are thought to have diverged first, while Crustacea form a sister clade to the Hexapoda (38) (Fig. 4 *A*, *Inset*). We identified two viruses associated with centipedes (Myriapoda) that fell as sister taxa to Hexapoda- and Crustacea-associated viruses (Fig. 4*A*). Viruses associated with the same host genera within the Hexapoda were generally more closely related, suggesting limited recent cross-species transmission. For example, mosquito-associated viruses largely clustered together (Fig. 4*A*, mosquito icon).

In contrast, the organization of clade 2 is consistent with a history of host jumping. Segments from five Crustacea-associated viruses (two crab-associated, and one each associated with lobster, shrimp and Amphipoda) formed a sister clade to Chelicerata-associated quaranjaviruses, and this relationship was observed in multiple segments (Fig. 4 *A*–*D*). The only exception was the HA phylogeny, in which the Crustacea-associated viruses were dispersed throughout the tree (Fig. 4*E*). However, due to the limited availability of HA segments in public datasets, this placement is likely to be an artifact of undersampling. The topology of this clade is also inconsistent with codivergence: Rather, it is suggestive of substantial host jumping. The ability to jump between diverse host species is apparent in the canonical quaranjaviruses (Quaranjfil quaranjavirus, Johnston Atoll quaranjavirus), as evidenced by the ability of these viruses to infect vertebrate and invertebrate hosts (Fig. 4*A*, “canonical quaranjaviruses”).

The placement of “Cheliceravirus” was inconsistent among segments. Both the PB1 and PB3 segments form a sister clade to the rest of the family with high support (Fig. 4 *A* and *C*). Few PB2 segments could be recovered from this putative genus. In contrast, the NP segments were more closely related to the Crustacea- and other Chelicerata-associated segments, while most of the HA segments cluster with the *Thogotovirus* outgroup. However, this phylogeny may change with the addition of more sequences.

## Discussion

We present evidence that the order *Articulavirales* originated in aquatic ecosystems and should be reorganized into four families. We document evidence of Cnidaria-associated *Articulavirales*, demonstrating that viruses in this order are likely able to infect ancient invertebrates. Our study also identifies an arachnid-associated genus in the “Quaranjaviridae”, which we have termed “Cheliceravirus”, and supports the addition of a new genus, denoted the “Metamyxovirus”, that appears to infect both vertebrate and invertebrate hosts. Finally, we greatly expanded the number of known quaranja-like viruses and propose that this genus be reclassified as a family—the “Quaranjaviridae”.

Although accurately rooting the phylogenetic tree of the *Articulavirales* is challenging, it is possible that ancient invertebrate lineages rather than fish are the first hosts of the *Articulavirales* for two reasons. First, this topology avoids introducing arguably unlikely vertebrate-to-invertebrate transitions. Second, rooting the tree with the *Amnoonviridae* contradicts some theories of virus genomic architectural evolution (9). If the *Amnoonviridae* is the root of the tree the most parsimonious host progression places fish as the first hosts of the *Orthomyxoviridae*. The characterized segments of Tilapia lake virus (PB1) and of salmon isavirus (PB1, PB2, and PB3) are approximately 50nt to 300nt shorter than their counterparts in the *Orthomyxoviridae*. This relationship implies that *Articulavirales* segments have increased in length since the genesis of the order. However, it has been hypothesized that viral segments become shorter and more streamlined over time (9). Moreover, the PB2 segment of the novel sturgeon- and carp-associated influenza-like viruses are longer than the PB2 segments from descendant taxa in the clade, implying a lengthening and then shortening of this segment. Thus, we argue that the currently available *Articulavirales* phylogeny should be rooted on the “Cnidenomoviridae”. Importantly, however, both tree topologies support an aquatic origin of the *Articulavirales* and indicate that this order may have persisted since the split of Anthozoa and Medusozoa Cnidaria in the Ediacaran (~640 Mya) or the evolution of fish, which is thought to have occurred at approximately the same time.

Consideration of the *Articulavirales* as a whole highlights that viruses within this order utilize a large repertoire of transmission routes. Influenza virus spreads via respiratory droplets in mammals and feces among birds. Fish-to-fish influenza transmission may also be respiratory, as fish- and amphibian-associated influenza-like viruses have been detected in gill tissue (23). There is evidence that both salmon isavirus and TiLV can be transmitted vertically (39, 40) as well as horizontally, potentially through an oral-fecal route (41). Characterising the transmission dynamics of the “Cnidenomoviridae” between basal invertebrates is beyond the scope of this study, but previous studies have suggested that crustaceans could serve as vectors for aquatic RNA viruses (42). Invertebrate-to-vertebrate transmission facilitated by terrestrial arthropods has evolved independently at least twice within the *Articulavirales* (“Quaranjaviridae” and *Thogotovirus*), and motile animals would have been necessary if the *Articulavirales* first emerged in sessile invertebrates such as corals. Crustaceans could play such a role, but fish are also plausible vector candidates because they can travel long distances and serve as hosts for at least two *Articulavirales* families. Answering this question and elucidating the mechanism by which the “Quaranjaviridae” spread among nonvertebrate biting arthropods will require experimental data but could shed light on mechanisms of cross-species transmission.

Our findings support the hypothesis that fish were early, if not the first, hosts of influenza-like viruses. This hypothesis was originally put forward after the discovery of a divergent influenza-like virus in a hagfish, although based on an assumption of virus–host codivergence (12, 43). The presence of divergent influenza-like viruses in a hagfish, carp, and sturgeon suggests that these viruses have likely existed throughout the entire evolutionary history of fish. Cyclostomata, to which Myxini (including hagfish) belong, is thought to have diverged from other vertebrate phyla around 600 Mya (44), suggesting that *Articulavirales* adapted to vertebrate hosts early in its evolutionary history. No influenza-like viruses have been detected in invertebrates. Although substantial sampling gaps persist, the discovery of other *Articulavirales* in a wide range of invertebrate hosts suggests that influenza viruses, as defined as a distinct phylogenetic group, are limited to vertebrate hosts, and implies that fish were the first. A similar evolutionary history has been proposed for the vertebrate-infecting family *Coronaviridae* (45). Amphibians form an evolutionary lineage between fish and reptiles and bridge aquatic and terrestrial ecosystems. The relative placement of amphibians is consistent with both virus–host codivergence and an aquatic-to-terrestrial transition. We find that the latter is more strongly supported by the phylogeny of the influenza clade. Taken together, the evolution of influenza viruses may therefore have been shaped by cross-class host jumps. Some events of this kind are still observable today [e.g., bird-to-mammal transmission of H5N1 (46)], while others, like the hypothetical transmission of influenza-like viruses between fish and other aquatic vertebrates, represent deep evolutionary relationships.

Our study places vector-borne virus emergence into an evolutionary context. In the family “Quaranjaviridae”, only arachnid-associated viruses are known to spill over into vertebrates. While this could be due in part to the biting behavior of ticks, the “Quaranjaviridae” have also been detected in mosquito genera known to transmit RNA viruses to humans (*Aedes* and *Culex*). Mosquito-borne transmission of quaranjaviruses to humans has not been reported. Although it may be that cryptic transmission does occur, as vertebrates and particularly humans are disproportionately oversampled, these events are likely to be captured if they are associated with overt disease. Thus, of the possible explanations for this ostensible absence, we consider two to be of particular interest: Spill over of mosquito-borne quaranjaviruses does occur but does not cause disease, or spill over of these viruses into vertebrates does not occur because of genetic differences between mosquito- and tick-borne “Quaranjaviridae”. If the former, it may be that these viruses are unable to evade the vertebrate adaptive immune system. If the latter, they may not be able to adapt to vertebrate host receptors. In either case, the absence of mosquito-borne quaranjavirus conferred disease suggests that there are key biological differences between Hexapoda-borne and Chelicerata-borne quaranjaviruses, and the latter are better able to adapt to vertebrate hosts. Elucidating these differences will require experimental data and could have important implications for understanding vector-borne virus emergence in vertebrates. The close relationship between arachnid- and crustacean-associated “Quaranjaviridae” again indicates that the evolutionary history of the *Articulavirales* has not followed strict virus–host codivergence. Chelicerata diverged prior to both Hexapoda and Crustacea (38), yet Crustacea-associated viruses are more closely related to Chelicerata-associated viruses and are basal to the rest of the family. Sampling horseshoe crabs, which are ancient, aquatic Chelicerata, could provide insight into how this transition occurred.

This study was not without limitations. We utilized publicly available data for most of our analyses, introducing sampling biases (e.g., overrepresentation of Arthropoda hosts) and limiting the size of our dataset. As such, the range of hosts of the *Articulavirales* is likely far larger than what we have described. Without experimental data, we cannot definitively establish the true hosts of the viruses we have identified; however, there is sufficient contextual data to draw reasonable inferences. We were unable to obtain access to the raw sequencing reads of the sturgeon library (TSA ID GIPE01): The reported SRAs did not match the assembled contigs available through the TSA database. As a consequence, we could not assess the abundance of the sturgeon influenza-like virus. Additionally, the use of cross-sectional metagenomic data (i.e., data collected at a single time point) precludes the assignment of true virus–host associations. Despite this, the host associations we assumed were supported by phylogenetic analysis. For example, viruses identified in *Heliopora* corals clustered together, as do most mosquito-associated “Quaranjaviridae”. Finally, while the overall topology of the phylogenies we inferred had strong support, internal tree nodes were less robust. This may reflect substantial undersampling within the order, and the addition of more viruses when discovered should improve the veracity of the placement of internal nodes. For this reason, we avoided overly interpreting internal relationships, which limited the depth of our conclusions.

We have demonstrated that metagenomic data can be used to elucidate the origins of respiratory and arthropod-borne viruses that are the focus of modern public health initiatives. Continuing to explore virus diversity in a wide range of animal hosts will help to answer key evolutionary questions. For example, how did the evolution of the adaptive immune system influence virus evolution, particularly when ancient invertebrate viruses first appeared in vertebrates? Answering this and related questions will be especially useful for improving methods to estimate zoonotic risk. Importantly, our findings show that, as one of the first animals to evolve on Earth and therefore one of the likely first animal hosts of RNA viruses, aquatic Metazoa have played a central role in shaping the modern virome. Further exploration of marine virus diversity will undoubtedly yield deeper insights into the drivers of virus diversity and shed light on how these simple replicating entities have successfully parasitized all known forms of life.

## Methods

### Sample Collection.

All (n = 128) coral samples were collected and identified by Zoe Richards. Location details for the two individual corals in which we identified novel *Articulavirales* sequences are as follows: *Heliopora coerulea* #210, WAM Z42167, subtidal 12m, collected 20.09.2016, NW Montelivet Island, Bonaparte Archipelago, inshore Kimberley, Western Australia. Site 196; S14.28778, E125.2127. *Acropora samoensis*, WAM Z421003, #158, intertidal, collected 18.09.2016, North Patricia Island, Bonaparte Archipelago, inshore Kimberley, Western Australia. Site 189S14.266603, E125.29700.

### RNA Extraction and Sequencing.

The first coral *(Heliopora coerulea*) was processed in 2018. The sample was thawed on ice, removed from RNA later, and rinsed gently with sterile RNA and DNA-free 1× PBS solution (GIBCO). Sterile tools were then used to remove a small fragment (~1 g), with care taken to include both soft and hard tissues. The tissue was homogenized using a sterile drill, and total RNA was extracted with the RNeasy Plus Mini Kit (Qiagen). RNA quality was determined using Bioanalyzer fragment analysis, followed by Ribo-Zero Plus library preparation and next-generation sequencing (Illumina HiSeq 2500) performed at the Australian Genome Research Facility (AGRF).

The second coral (*Acropora samoensis*) was processed in 2022. This sample was homogenized in liquid nitrogen using a mortar and pestle. RNA was extracted using the RNeasy Plus Mini Kit (Qiagen). The quality and concentration of the RNA were assessed with Qubit. Ribo-Zero Plus library preparation and next-generation sequencing (Illumina NovaSeq S4) were performed at AGRF. Negative extraction controls were used to assess potential contaminants. No relevant contaminants were identified. The extracted RNA from each sample was not pooled prior to sequencing.

### Virus Discovery.

Raw sequencing reads were trimmed using Trimmomatic v0.38 (47). Reads mapping to rRNA were removed using sortMeRNA v4.3.3 (48). Contigs were assembled from trimmed and filtered reads using MEGAHIT v1.2.9 (49). Assembled reads were screened against the NCBI nonredundant (nr) protein database (as of June 2022) and a custom RdRp database using Diamond BLASTx v.2.0.9. The custom RdRp database contains all currently identified RdRp sequences. In both cases, we used an e-value cutoff of 1 × 10^−5^. Contigs with hits to known viruses with less than 80% amino acid identity were considered new virus species. All contigs with hits to the RdRp database were cross-checked against the nr results, and virus candidates that hit to host genes were excluded from further analysis. Contigs identified as *Articulavirales* segments were translated using the Expasy online translation tool (https://web.expasy.org/translate/), and each translation was visually inspected in Geneious. Read abundance was estimated using RSEM v.1.3.0 (50) implemented in Trinity v.2.5.1 (51).

A structure-based approach was adopted to search for additional polymerase segments in the coral libraries. All contigs of at least 1200nt in length that did not correspond to an entry in the RdRp or nr databases were translated using EMBOSS. Contigs with open reading frames between 600 and 900 amino acids were retained. This range was selected according to the range in lengths of known *Articulavirales* polymerase segments. The remaining contigs were analyzed using Phyre2 to identify structural similarities to known *Articulavirales* polymerase segments.

### Assessment of Endogenous Virus Elements (EVEs).

All virus candidates were compared to the nr database (as of June 2022) to confirm that they did not share similarity to known host genes. We also assessed the presence of host gene contamination using the contamination function implemented in CheckV (29). No virus candidates were found to be EVEs.

### NCBI TSA Database Screen.

We performed tBLASTn searches limited to the following organisms: Ascidiacea, Porifera, Cnidaria, Arthropoda, Bryozoa, Mollusca, fish (Agnatha, Chondrichthyes, Actinopterygii, Osteichthyes actinopterygian, Osteichthyes coelacanthiform, Osteichthyes dipnoan, Hyperoartia, Gnathostomata, Petromyzontidae, marine lamprey, Arctic lamprey), and Amphibia. These organisms encompass the majority of aquatic animals with available TSA libraries. To identify divergent PB1 segments, we began by using the Wenling hagfish influenza-like virus (AVM87635), Beihai orthomyxo-like virus 2 (APG77864) as input, as well as both PB1 segments identified in corals. Divergent hits (less than 80% amino acid identity) were confirmed to be novel using BLASTx (nr database). We used these sequences as input for tBLASTn against the TSA projects described above. Each novel sequence was translated using Expasy and visually inspected in Geneious. In all cases, only translations containing the RdRp SDD motif that defines the *Articulavirales* were considered. A preliminary phylogenetic analysis was performed to remove identical sequences. In this case, sequences were aligned in MAFFT v7.490 (33), and the phylogenetic tree was inferred using the maximum likelihood approach in IQ-TREE v1.6.12 (31) with ModelFinder, which selected LG+F+R10 as the best-fit model.

### Serratus Screen.

To identify contigs assembled as part of the Serratus project (27) containing “novel” or unassigned *Orthomyxoviridae* PB1 segments, we downloaded and manually queried the master RdRp contig and metadata file for *Orthomyxoviridae*-like PALMdb palmprint (or barcode) accessions (n = 336). These PALMdb palmprints are assigned based on taxonomy and clustered based on 90% identity (52). Putative novel Orthomyxo-like PB1 sequences were manually validated using BLASTx against the NCBI nr virus database. SRA accessions from identified novel viral-like sequences were assembled using MEGAHIT, and additional segments were extracted from assemblies using tBLASTn.

### Phylogenetic Tree Estimation.

To compile a representative dataset of publicly available *Articulavirales* PB1 segments, we downloaded all noninfluenza and influenza-like *Articulavirales* PB1 segments available on NCBI “Virus” that were at least 500 amino acids in length (n = 291). For completeness, we used all available spellings of “PB1” and “polymerase basic 1.” Sequences associated with a laboratory host were excluded. We included all influenza-like viruses identified in nonmammalian and nonreptilian hosts (n = 8) and a representative sample of influenza A, B, C, and D viruses (n = 22). Identical sequences were removed using CD-HIT v4.6.1 with a threshold of 0.99. The remaining sequences were aligned with MUSCLE v5.1 (30). After trimming the aligned sequences to the conserved motifs in Geneious and removing gaps with trimAl v1.4 (53), identical sequences were again removed according to genetic distance. The final dataset contained 288 sequences.

Sequences for all other phylogenies presented in this study were aligned using MUSCLE v5.1 unless otherwise specified. In all cases, gaps were removed using trimAl v1.4., and maximum likelihood phylogenetic trees based on amino acid sequences were inferred in IQ-TREE v1.6.12 using 1000 ultrafast bootstraps and ModelFinder (Table 1). Trees were rendered with ggtree (54) implemented in R v4.1.2 and visualized with Adobe Illustrator.

**Table 1.** Substitution models selected by Model Finder during phylogenetic inference

Phylogenetic tree	Figure	Substitution model
*Articulavirales* PB1	2 *A*–*C*	LG+F+R10
Influenza clade PB1	3*A*	LG+F+R10
Influenza clade PB2	3*B*	LG+F+R3
Influenza clade PB3/PA	3*C*	LG+F+Γ4
Quaranjaviridae PB1	4*A*	LG+F+R10
Quaranjaviridae PB2	4*B*	LG+F+R6
Quaranjaviridae PB3	4*C*	LG+F+R9
Quaranjaviridae NP	4*D*	LG+F+R10
Quaranjaviridae HA	4*E*	WAG+F+R6

### Analysis of Host Gene Distribution in Sequencing Libraries.

We analyzed the host gene assignment and distribution of the sequencing libraries associated with the Siberian sturgeon (*Acipenser baerii*, TSA: GIPE01, BioProject: PRJNA591120) as well as the corals from which we extracted RNA (*Acropora samoensis* and *Heliopora coerulea*). For the sturgeon library, the transcriptome shotgun contigs preassembled and associated with the BioProject ID were used as input. In all three cases, we used KMA v1.3.9a (34) and CCMetagen v1.1.3 (35) and visualized the results with Prism v9.5.0 and Adobe Illustrator.

## Supplementary Material

Appendix 01 (PDF)

Dataset S01 (XLSX)

Dataset S02 (XLSX)

Dataset S03 (XLSX)

## Data Availability

Sequence data for the two viruses identified through metatranscriptomic sequencing (Heliopora cnidenomovirus 1 and Acropora cnidenomovirus 1) have been submitted to both the SRA (BioProject PRJNA966762) (55) and GenBank (accessions OQ939982.1 and OQ939981.1, respectively). The sequence accessions for the viruses identified here through data mining are provided in *SI Appendix*. The multiple sequence alignments used to generate all of the phylogenetic trees presented in this study are freely available at https://github.com/mary-petrone/ancient_articulavirales (56).
