# Supplementary material for: Evidence for an ancient aquatic origin of the RNA viral order Articulavirales
Source: Proc Natl Acad Sci U S A. 2023 Oct 31;120(45):e2310529120. doi: 10.1073/pnas.2310529120 (PMC10636315; doi:10.1073/pnas.2310529120)
Supplement: Supplementary file 1 — Appendix 01 (PDF) [file pnas.2310529120.sapp.pdf]

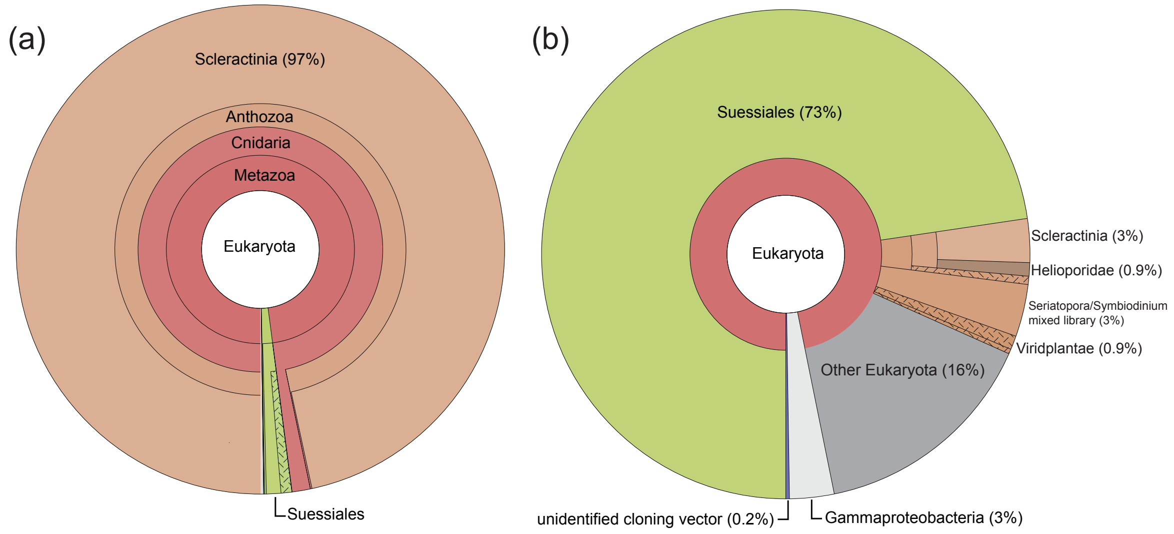


**Figure S1. Composition of coral sequencing libraries derived using KMA and CCMetagen.** (a) *Acropora samoensis* library (b) *Heliopora coerula* library

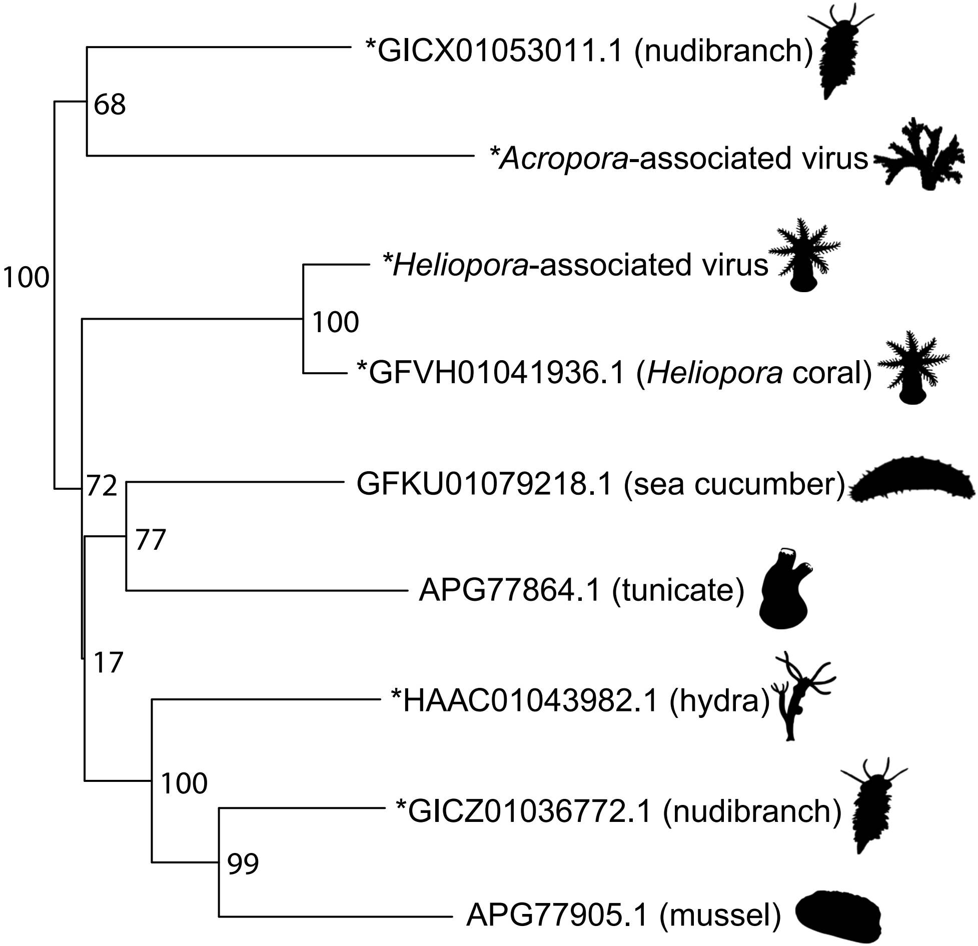


**Figure S2. Composition of the known *Cnidenomoviridae*.** Tips are labeled with putative host. Branch lengths are scaled to the number of amino acid substitutions per site. *Denotes virus identified in the study.

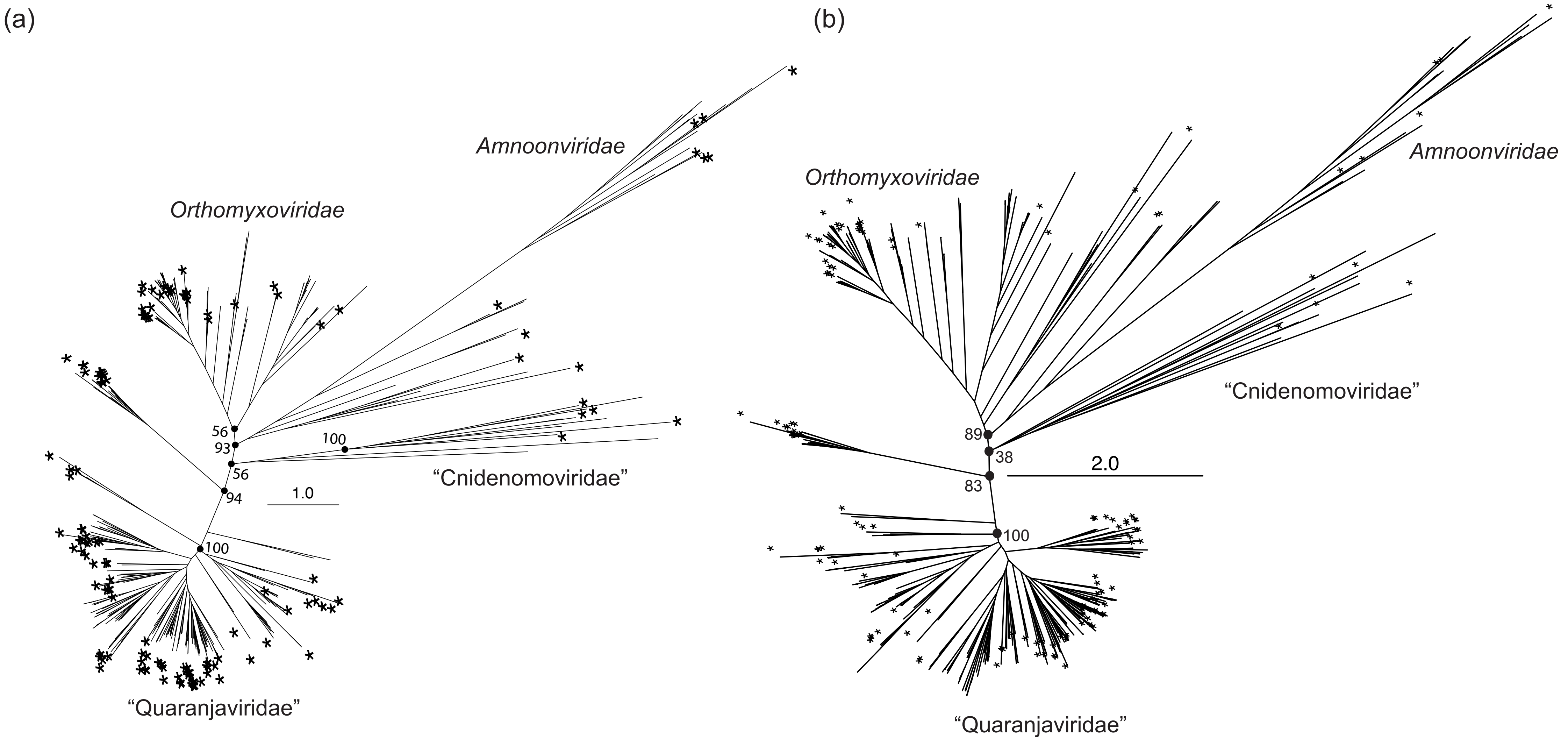


**Figure S3: Phylogenetic reconstruction of the *Articulavirales* using (a) MUSCLE and (b) MAFFT aligners.** Branch lengths are scaled to the number of amino acid substitutions per site.

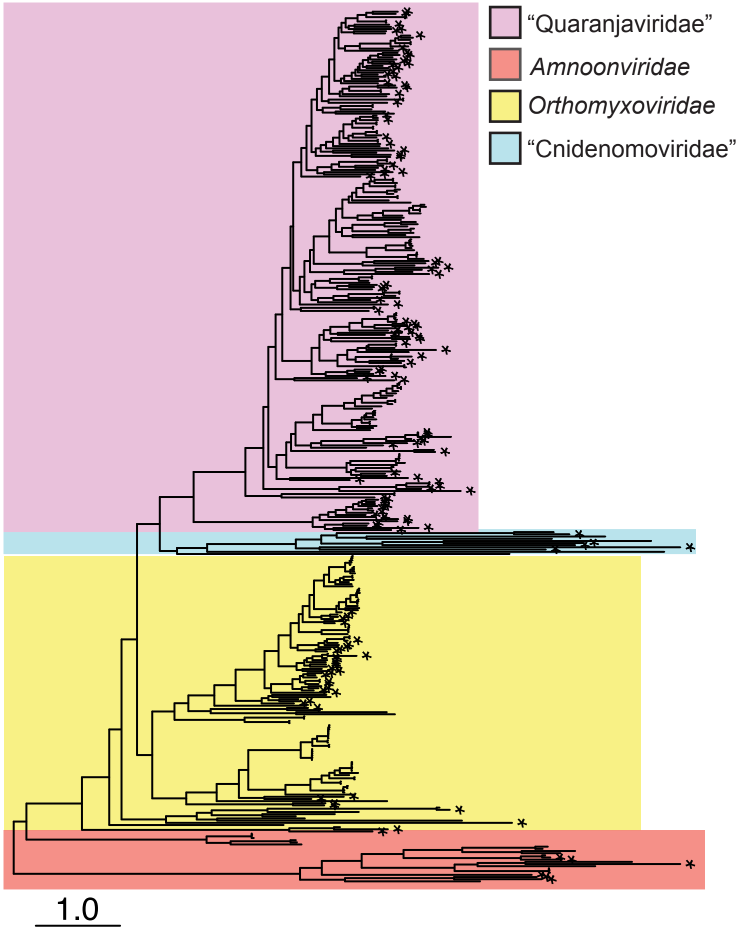


**Figure S4: Midpoint rooted phylogeny of the *Articulavirales*.** Branch lengths are scaled to the number of amino acid substitutions per site. *Denotes viruses identified in this study.

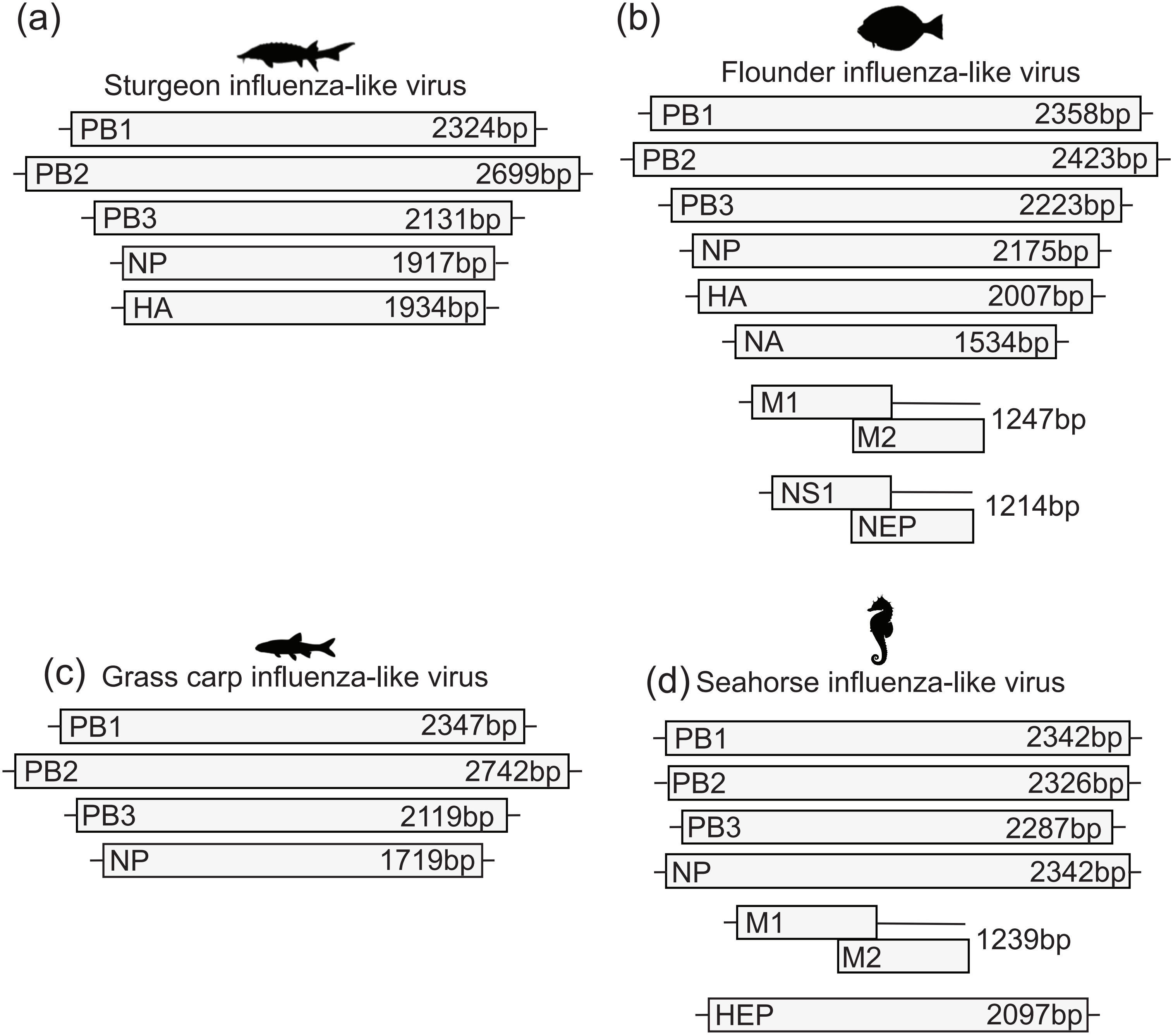


**Figure S5: Segments of novel influenza-like viruses discovered in fish.**

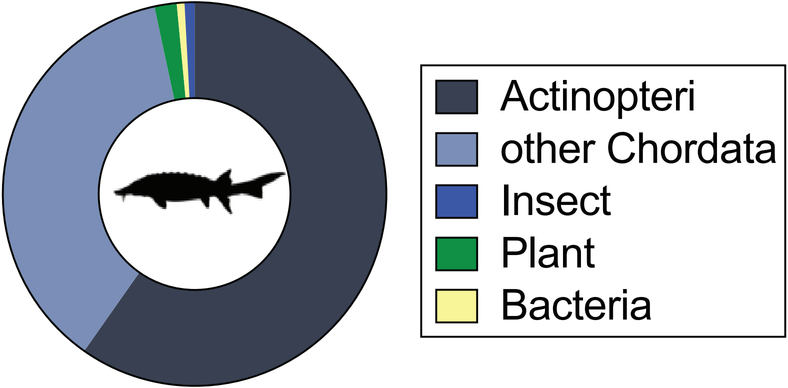


**Figure S6: Composition of *Acipenser baerii* (TSA ID: GIPE01) sequencing library derived using KMA and CCMetagen**

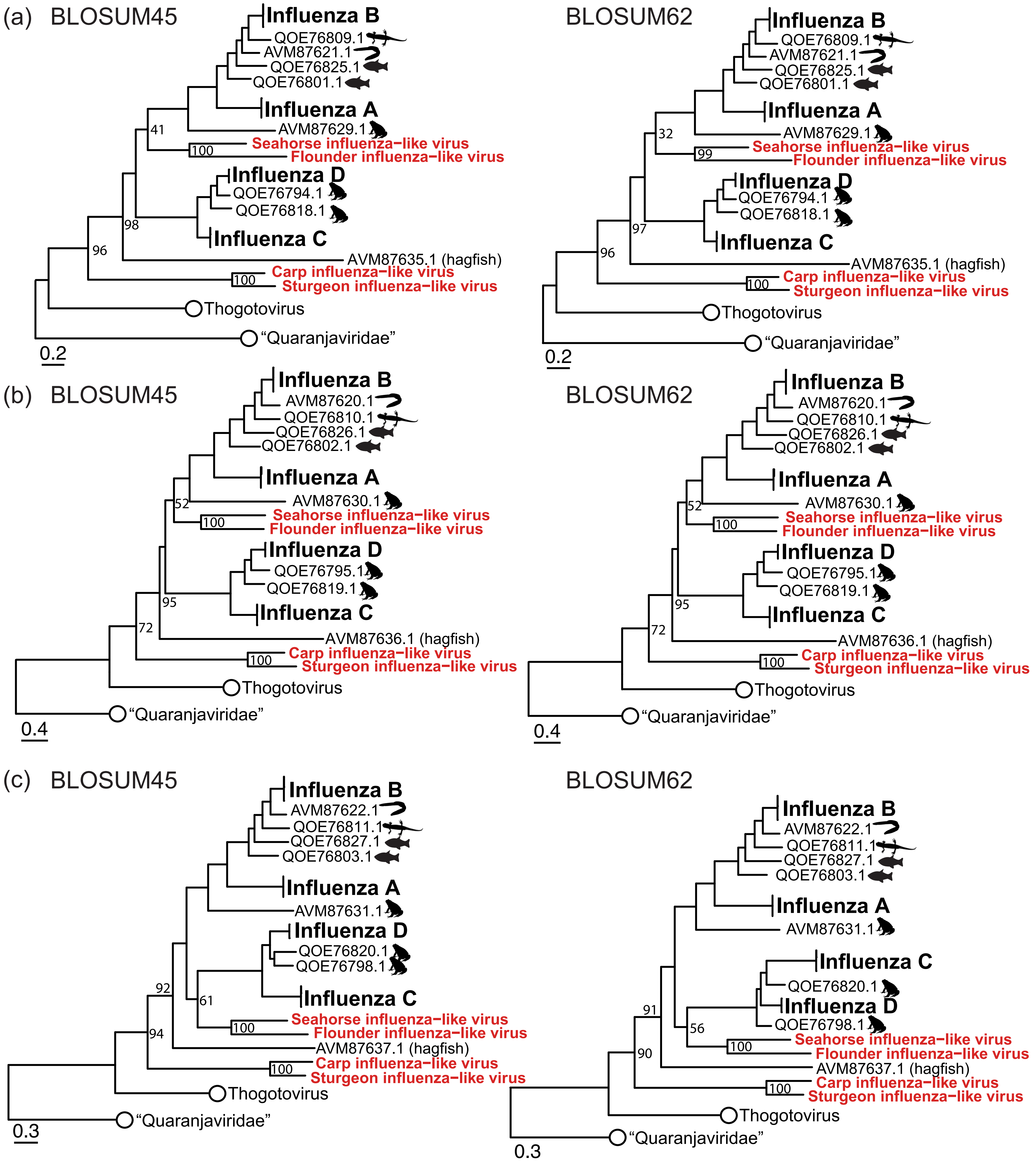


**Figure S7: Phylogenetic inference of the influenza clade using MAFFT aligner**. UFBoot values are shown at key nodes. Branches are scaled to amino acid substitutions. (a) PB1, (b) PB2, (c) PB3

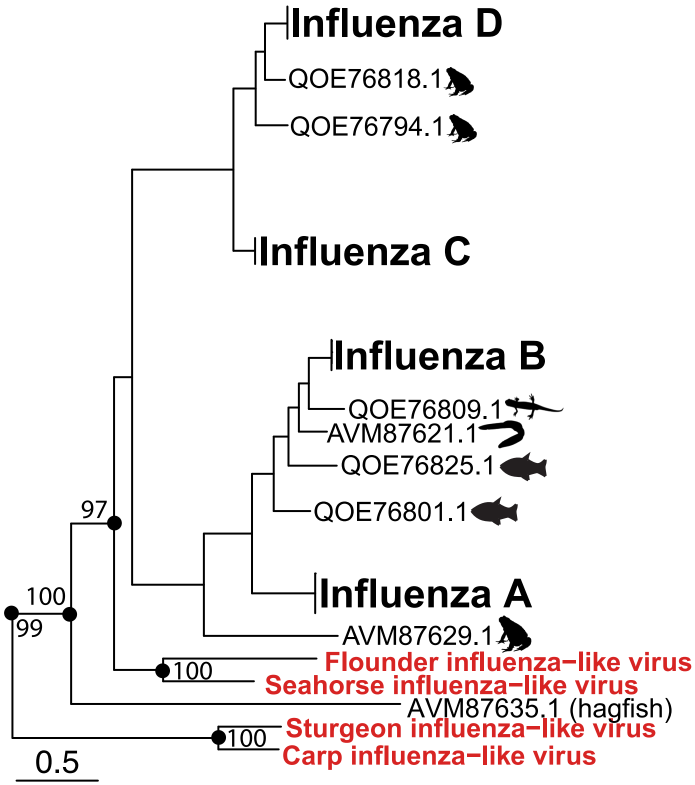


**Figure S8: Influenza clade extracted from MUSCLE-aligned *Articulavirales* phylogenetic tree (Fig. 2).**
Branches are scaled to amino acid substutions.

**Table S1. Summary of BLAST results for novel coral-associated *Articulavirales*.** nr: non-redundant protein database (NCBI), custom: custom RdRp database

| Host species | BLAST (nr/custom) | % identity (nr/custom) | e-value (nr/custom) |
|---|---|---|---|
| *Heliopora coerulea* | PB1, Beihai orthomyxo-like virus 2/PB1, Ornate chorus frog influenza-like virus | 22.6/23.7 | 7.14e-15/2.66e-14 |
| *Acropora samoensis* | PB1, Soybean thrips quaranja-like virus 2/none | 25.2/none | 9.18e-06 |
